# Circulating cytokine levels and 5-year vascular recurrence after stroke: A multicenter prospective cohort study

**DOI:** 10.1093/esj/23969873251360145

**Published:** 2026-01-01

**Authors:** Lanyue Zhang, Mohamad Ali Antabi, Jana Mattar, Omar El Bounkari, Rong Fang, Karin Waegemann, Felix J Bode, Sebastian Stösser, Peter Hermann, Thomas G Liman, Christian H Nolte, Benno Ikenberg, Kathleen Bernkopf, Wenzel Glanz, Daniel Janowitz, Annika Spottke, Michael Wolfgang Görtler, Silke Wunderlich, Inga Zerr, Gabor C Petzold, Matthias Endres, Jürgen Bernhagen, Martin Dichgans, Marios K Georgakis, DEMDAS Investigators, Matthias Endres, Emrah Duezel, Lucia Kerti, Katja Neumann, Julius N Meißner, Thomas G Liman, Lucia Kerti, Christian H Nolte, Tatjana Wittenberg, Jan F Scheitz, Harald Prüß, Pia Sophie Sperber, Alexander H Nave, Anna Kufner Ibaroule, Gabor Petzold, Felix Bode, Sebastian Stösser, Julius Meissner, Taraneh Ebrahimi, Julia Nordsiek, Niklas Beckonert, Christine Kindler, Inga Zerr, Peter Hermann, Matthias Schmitz, Stefan Goebel, Timothy Bunck, Julia Schütte-Schmidt, Sabine Nuhn, Corinna Volpers, Peter Dechent, Mathias Bähr, Michael Görtler, Wenzel Glanz, Valentina Perosa, Martin Dichgans, Frank Wollenweber, Marios Georgakis, Rong Fang, Daniel Janowitz, Karin Waegemann, Steffen Tiedt, Silke Wunderlich, Benno Ikenberg, Kathleen Bernkopf, Christiane Huber, Holger Poppert, Marco Düring, Miguel Ángel Araque Caballero, Benno Gesierich, Anna Dewenter, Laura Dobisch, Katja Neumann, Oliver Speck, Annika Spottke, Tony Stöcker, Peter Bartenstein, Michael Wagner

**Affiliations:** Institute for Stroke and Dementia Research (ISD), LMU University Hospital, LMU Munich, Munich, Germany; Institute for Stroke and Dementia Research (ISD), LMU University Hospital, LMU Munich, Munich, Germany; Institute for Stroke and Dementia Research (ISD), LMU University Hospital, LMU Munich, Munich, Germany; Institute for Stroke and Dementia Research (ISD), LMU University Hospital, LMU Munich, Munich, Germany; Institute for Stroke and Dementia Research (ISD), LMU University Hospital, LMU Munich, Munich, Germany; Institute for Stroke and Dementia Research (ISD), LMU University Hospital, LMU Munich, Munich, Germany; German Center for Neurodegenerative Diseases (DZNE), Munich, Germany; German Center for Neurodegenerative Diseases (DZNE), Bonn, Germany; Department of Vascular Neurology, University Hospital Bonn, Bonn, Germany; German Center for Neurodegenerative Diseases (DZNE), Bonn, Germany; Department of Vascular Neurology, University Hospital Bonn, Bonn, Germany; Department of Neurology, University Medical Center Göttingen, Göttingen, Germany; German Center for Neurodegenerative Diseases (DZNE), Göttingen, Germany; Center for Stroke Research Berlin (CSB), Charité – Universitätsmedizin Berlin, Berlin, Germany; German Center for Neurodegenerative Diseases (DZNE), Berlin, Germany; Department of Neurology, Carl Von Ossietzky University, Oldenburg, Germany; German Center for Neurodegenerative Diseases (DZNE), Berlin, Germany; Berlin Institute of Health (BIH), Berlin, Germany; Department of Neurology with Experimental Neurology, Charité – Universitätsmedizin Berlin, Berlin, Germany; Department of Neurology, Klinikum rechts der Isar, School of Medicine and Health, Technical University of Munich, Munich, Germany; Department of Neurology, Klinikum rechts der Isar, School of Medicine and Health, Technical University of Munich, Munich, Germany; Department of Neurology, University Hospital, Otto-von-Guericke University Magdeburg, Magdeburg, Germany; German Center for Neurodegenerative Diseases (DZNE), Magdeburg, Germany; Institute for Stroke and Dementia Research (ISD), LMU University Hospital, LMU Munich, Munich, Germany; German Center for Neurodegenerative Diseases (DZNE), Bonn, Germany; Department of Vascular Neurology, University Hospital Bonn, Bonn, Germany; Department of Neurology, University Hospital, Otto-von-Guericke University Magdeburg, Magdeburg, Germany; German Center for Neurodegenerative Diseases (DZNE), Magdeburg, Germany; Department of Neurology, Klinikum rechts der Isar, School of Medicine and Health, Technical University of Munich, Munich, Germany; Department of Neurology, University Medical Center Göttingen, Göttingen, Germany; German Center for Neurodegenerative Diseases (DZNE), Göttingen, Germany; German Center for Neurodegenerative Diseases (DZNE), Bonn, Germany; Department of Vascular Neurology, University Hospital Bonn, Bonn, Germany; Center for Stroke Research Berlin (CSB), Charité – Universitätsmedizin Berlin, Berlin, Germany; German Center for Neurodegenerative Diseases (DZNE), Berlin, Germany; Department of Neurology with Experimental Neurology, Charité – Universitätsmedizin Berlin, Berlin, Germany; German Centre for Cardiovascular Research (DZHK), Partner Site Berlin, Berlin, Germany; German Center for Mental Health (DZPG), Partner Site Berlin, Berlin, Germany; Institute for Stroke and Dementia Research (ISD), LMU University Hospital, LMU Munich, Munich, Germany; German Centre for Cardiovascular Research (DZHK), Munich, Germany; Munich Cluster for Systems Neurology (SyNergy), Munich, Germany; Institute for Stroke and Dementia Research (ISD), LMU University Hospital, LMU Munich, Munich, Germany; German Center for Neurodegenerative Diseases (DZNE), Munich, Germany; German Centre for Cardiovascular Research (DZHK), Munich, Germany; Munich Cluster for Systems Neurology (SyNergy), Munich, Germany; Institute for Stroke and Dementia Research (ISD), LMU University Hospital, LMU Munich, Munich, Germany; Munich Cluster for Systems Neurology (SyNergy), Munich, Germany; Program in Medical and Population Genetics and Cardiovascular Disease Initiative, Broad Institute of MIT and Harvard, Cambridge, MA, USA

**Keywords:** Stroke, recurrence, inflammation, cytokines, risk prediction

## Abstract

**Background and objectives:**

Anti-inflammatory therapies are tested in randomized trials for secondary stroke prevention. Detecting inflammatory biomarkers that predict vascular recurrence could optimize patient selection for these trials.

**Methods:**

In a multicenter prospective cohort study, we measured plasma levels of 22 inflammatory cytokines in 486 acute stroke patients (474 ischemic strokes and 12 intracerebral hemorrhages; median age 68 years, 34% female, median 3 days post-stroke onset). Patients were followed for over 5 years through telephone and in-person interviews to record the occurrence of the following outcomes: (1) recurrent stroke or transient ischemic attack (TIA; primary outcome); (2) a composite of recurrent vascular events (stroke, TIA, acute coronary syndrome, hospital admission due to heart failure, and death; secondary outcome). Associations between cytokine levels and these outcomes were analyzed using Cox proportional hazards models adjusted for demographic and vascular risk factors.

**Results:**

During the 5-year follow-up period, 59 patients (12.1%) experienced recurrent stroke or TIA, and 118 (24.3%) experienced recurrent vascular events. After adjustments for demographic and vascular risk factors, and correction for multiple comparisons, higher plasma levels of CD62E (adjusted Hazard Ratio (aHR)/SD increment: 1.63, 95%CI 1.22–2.20) and MIF (aHR: 1.56, 95%CI 1.18–2.06) in the acute phase after stroke were statistically significantly associated with increased risk of recurrent stroke or TIA. The associations followed a dose-response pattern across quartiles of CD62E and MIF levels. Adding baseline CD62E and MIF levels to models including age, sex, vascular risk factors, and baseline C-reactive protein (CRP) levels led to significant improvements in the prediction of 5-year risk of recurrent stroke or TIA (ΔC-index 0.030–0.050).

**Conclusion:**

Among stroke patients, higher baseline levels of CD62E and MIF improved prediction of 5-year risk of recurrent stroke or TIA on top of vascular risk factors and CRP levels. Whether assessment of these cytokines could improve patient selection for secondary prevention trials of anti-inflammatory treatments, should be explored in future studies.

## Graphical Abstract

**Graphical abstract:**
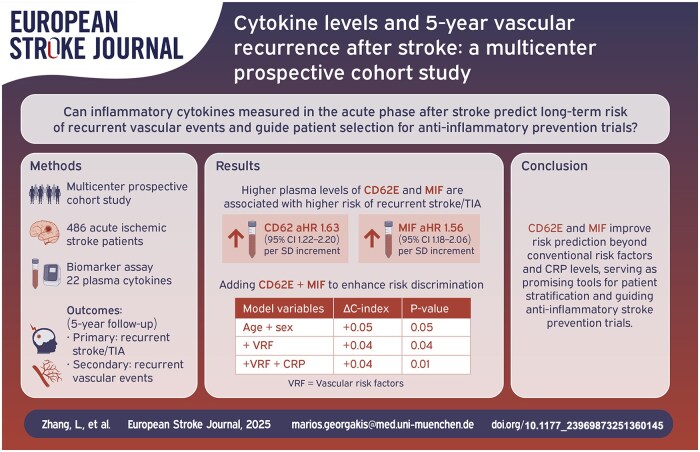

## Introduction

Stroke is one of the leading causes of mortality and adult disability worldwide. In 2021, there were 93.8 million recorded prevalent strokes, accounting for 7.3 million deaths and 160.5 million disability-adjusted life-years globally.^1^ Stroke prevalence is projected to increase from 3.9% to 6.4% among the U.S. adult population, posing a major public health burden.^2^ Despite advances in secondary prevention, stroke survivors face an alarmingly high risk of recurrent vascular events, with up to 40% encountering a new stroke, acute coronary events, or cardiovascular death within 5 years.^3–6^ These rates point to a high residual risk that current secondary prevention strategies fail to address.^7^

A growing body of evidence supports targeting inflammation as a potential strategy for secondary stroke prevention.^8–10^ The central role of inflammation in the progression of atherosclerotic cardiovascular diseases^11^ and stroke has been well documented through experimental research^12^, genetic epidemiology^13,14^, and vascular imaging studies.^15,16^ Moreover, three phase 3 trials (CANTOS,^17^ LoDoCo2,^18^ COLCOT^19^) provided evidence that anti-inflammatory treatment with canakinumab or colchicine reduces vascular events in patients with coronary artery disease. Aiming to translate this concept to secondary stroke prevention, the recently completed CONVINCE study which testing the non-specific anti-inflammatory drug colchicine revealed no significant reduction in recurrent vascular events among patients with a recent non-disabling non-cardioembolic stroke.^10,20^ However, notable risk reductions were observed in subgroups with atherosclerotic carotid stenosis or a history of coronary artery disease, underscoring the necessity for a more precise selection of patients who could benefit from anti-inflammatory therapies.

High-sensitivity C-reactive protein (CRP) and interleukin-6 (IL-6) are established inflammatory markers strongly associated with cardiovascular disease risk.^21,22^ Both biomarkers have been previously associated with the risk of incident^23^ and recurrent^24^ stroke. CRP has also been used to select patients for inclusion in trials testing anti-inflammatory agents for cardiovascular disease.^17,25^ However, their utility for secondary stroke prevention is debated due to high intra-patient variability, especially in the acute phase of stroke, and their lack of specificity for vascular inflammation.^26^ Moreover, both proteins are acute-phase reactants, and their levels might increase as a result of infections or systemic inflammatory conditions.^27^

In order to improve risk stratification and optimize patient selection for anti-inflammatory secondary preventive strategies, it is essential to identify and validate new biomarkers.^28^ Here, we performed an exploratory analysis within the multicenter prospective DEMDAS cohort – to investigate a literature-informed panel of 22 cytokines, chemokines and adhesion proteins as predictors of vascular recurrence. Specifically, we measured baseline plasma levels of these mediators in 486 patients shortly after stroke and explored associations with risk of recurrent stroke and recurrent vascular events over a follow-up period of 5 years.

## Methods

### Study design and baseline assessments

Participants were drawn from the DEMDAS study (DZNE (German Center for Neurodegenerative Diseases) – Mechanisms of Dementia After Stroke), a multicenter, prospective cohort study conducted at six tertiary stroke centers in Germany. Details on the study’s design, rationale, and baseline characteristics have been detailed elsewhere.^29,30^

In brief, DEMDAS recruited 600 patients aged 18 years or older who had experienced an acute stroke – defined as new focal neurological deficits lasting >24 h with imaging confirmation of either an acute ischemic infarct (via diffusion-weighted MRI lesion or new CT lesion) or an intracerebral hemorrhage – provided symptom onset occurred within the 5 days prior to admission, and who had no prior diagnosis of dementia. Exclusion criteria included severe comorbidities such as malignancies or end-stage renal disease, as well as the inability to provide consent. The recruitment period ran from May 2011 to January 2019. Participants underwent in-person follow-up assessments at 6-, 12-, 36-, and 60-months post-enrollment. This study adheres to the Strengthening the Reporting of Observational Studies in Epidemiology (STROBE) guidelines.^31^

Participants underwent detailed interviews using standardized questionnaires to collect sociodemographic details, current smoking and drinking status, medical, and family history. Medication data (antihypertensive, antiplatelet, anticoagulant agents, and statins) were recorded from hospital discharge documentation. Clinical assessments included body weight and height (body mass index (BMI) was calculated as weight in kilograms divided by the square of height in meters), and blood pressure measured as the average of two seated readings taken after 5 min of rest. Stroke severity was assessed using the NIH Stroke Scale (NIHSS), and ischemic stroke subtypes were classified according to the Trial of ORG 10172 in Acute Stroke Treatment (TOAST) criteria.^32^ LDL cholesterol and CRP were extracted from hospital records and analyzed in mg/dL. CRP values were ln-transformed prior to analysis. These baseline definitions are consistent with previous DEMDAS publications.^29,30,33,34^

### Standard protocol approvals and patient consent

The study was conducted in accordance with the Declaration of Helsinki and received ethical approval from institutional review boards of the participating sites. Primary ethics approval for the multicenter study was granted by the Ethics Committee of the Medical Faculty at LMU Munich (201-13) and additional approvals were obtained from the Ethics Committee of the Medical Faculty at the University of Bonn (116/13), the Ethics Committee of the University Medical Center Göttingen (21/1/12), the Ethics Committee of the Technical University of Munich (93/14 S), and the Ethics Committee of Otto von Guericke University at the Medical Faculty and University Hospital Magdeburg (66/13). The Berlin study site (Charité – Universitätsmedizin Berlin) was covered by the ethics approval of the Medical Faculty of LMU Munich, according to Section 15 of the Berlin Medical Association Professional Code of Conduct (September 2009). Written informed consent was obtained from all participants or their legal representatives.

### Blood sample collection and processing

Blood samples for the current analysis were collected at baseline (within 5 days of stroke onset). The collection included one 9 mL Serum-Monovette for serum, three 9 mL EDTA-Monovettes (one specifically designated for plasma and two for DNA isolation), and one 2.5 mL Qiagen PAXGene tube for RNA-stabilized whole blood. Samples were processed immediately at each site and subsequently stored in a centralized biobank at the Institute for Stroke and Dementia Research (ISD) in Munich. Samples were centrifuged at 2000*g* for 10 min at 15 °C, aliquoted into 300 µL aliquots, and stored at −80 °C. To minimize pre-analytical variability, rigorous quality control measures were implemented, including adherence to standard operating procedures and regular audits that have been previously described.^29^ The specimens were double-pseudonymized and recorded using a protected data integration system to maintain confidentiality and traceability.

### Cytokine quantification and quality control

The Luminex^®^ Procartaplex Multiplex Assay was used to quantify the levels of 22 cytokines in plasma baseline samples. The quantified cytokines included IP-10/CXCL10, MCP-1/CCL2, MIF, MIP-1α/CCL3, MIP-1β/CCL4, TNF-α, CD40L, CD62E/E-selectin, CD62P/P-selectin, CCL11, CSF-2, IFN-α, IFN-γ, IL-1α, IL-1β, IL-6, IL-4, IL-8/CXCL8, IL-10, IL-12p70, IL-13, and IL-17A. This panel was selected on the basis of previous evidence linking these cytokines to atherosclerosis and arterial inflammation.^35,36^ Plasma samples were thawed, mixed thoroughly, and prepared for analysis according to the manufacturer’s protocol. The Capture Bead Mix was vortexed for 30 s before adding 50 µL to each well of a 96-well plate. A Hand-Held Magnetic Plate Washer ensured complete beads washing. Standards were prepared using a four-fold serial dilution to generate a standard curve for accurate quantification. Initial tests determined that the optimal dilution factor for the plasma samples was 1:4, balancing sensitivity with the assay’s dynamic range. Plasma samples (25 µL) were added to the wells containing the Capture Bead Mix and incubated on a shaker at 600 rpm for 2 h at room temperature. The plate was analyzed using the Luminex 200 machine, and the results were processed using xPONENT v. 3.1 software (Luminex Corporation). The doublet discrimination (DD) gate was set at 7500–25,000, with a sample volume of 50 µL, and at least 50 events/bead were recorded. A final reading volume of 120 µL/well was achieved by calibrating the device to detect at least 50 beads/analyte/sample. The concentration of the analytes was calculated using ProcartaPlex Analysis App software (Invitrogen) with a five-parameter logistic (5PL) fitting model. Data were log-transformed and standardized by batch to minimize between-batch variability, achieved by subtracting batch-specific means and dividing by batch-specific SD of ln-transformed cytokine values. Intra-assay and inter-assay coefficients of variation were monitored to ensure data reliability. Out-of-range (OOR) measurements below the detection limit, accounting for 3.9% of the data, were assigned half the lowest detectable concentration for each cytokine. Measurements exceeding 5 SD above or below the mean were excluded as potential technical errors.

### Outcome assessment and follow-up

Follow-up assessments were conducted at 6, 12, 36, and up to 60 months and included clinical evaluations, imaging, and medical record reviews by study physicians. The primary outcome of the study was recurrent stroke or transient ischemic attack (TIA). Recurrent stroke was defined as the acute onset of new neurological symptoms consistent with stroke and confirmed by imaging (MRI or CT) as a new infarct or hemorrhage distinct from the initial event. TIA was defined as the acute onset of neurological symptoms that resolved within 24 h without imaging evidence of a new infarction. Outcome events were initially reported by participants or their informants during structured follow-up assessments. All reported events were subsequently verified by study physicians through thorough inspection of medical records, imaging studies, and available clinical documentation. For participants who missed scheduled follow-ups, a standardized contact protocol was implemented, including phone calls, mailed questionnaires, and registry office checks, to determine recurrence status.^29,33^ The secondary composite outcome included recurrent vascular events, defined as the occurrence of any of the following after the initial stroke: myocardial infarction, angina pectoris, hospitalization due to acute or decompensated heart failure, TIA, recurrent stroke, or all-cause mortality. All outcomes were confirmed by a thorough inspection of the participants’ medical records, review of imaging studies, and clinical assessments during the in-person follow-up interviews by the study physicians. Deaths from any cause were recorded throughout the follow-up period, with the cause determined through medical record reviews and, when necessary, by contacting family members or caregivers, or accessing records from local registration offices.

### Statistical analyses

All statistical analyses were performed using R (version 4.3.3). The following R packages were utilized: *survival* package (version 3.7-0) for survival analysis, *survminer* package (version 0.4.9) for creating Kaplan–Meier curves, *survcomp* package (version 1.52.0) for C-index calculation, survIDINRI package (version 1.1–2) to compute category free net reclassification index (cfNRI) and integrated discrimination improvement (IDI) and *ggplot2* (version 3.5.1) for data visualization. Baseline characteristics for continuous variables were expressed as medians with interquartile ranges (IQR) and for categorical variables as counts and percentages. For group comparisons, the Mann–Whitney *U* or the Kruskal–Wallis tests were used for continuous variables, and the χ^2^ or Fisher’s exact tests were used for categorical variables.

We assessed potential determinants of cytokine levels using univariable linear regression models, with ln-transformed levels of each cytokine as the dependent variable. Each cytokine was analyzed separately against each predictor variable to evaluate individual associations. predictor variables included demographic (age, sex, BMI), clinical (LDL cholesterol, hypertension, diabetes, history of atrial fibrillation or stroke, current smoking, and drinking), technical (year of recruitment, time from symptom onset to blood collection), and disease-specific factors (NIHSS score, infarct volume). *p* values from all cytokine–predictor tests were adjusted for multiple comparisons with the Benjamini–Hochberg false-discovery-rate (FDR) correction; associations with FDR-adjusted *p* < 0.05 were considered significant.

Cox proportional hazards regression models were employed to evaluate associations between cytokine levels and time-to-event outcomes, including primary outcomes of recurrent stroke/TIA and recurrent vascular events over a 5-year follow-up period. Ln-transformed cytokine levels were analyzed as both continuous (per SD increment) and categorical variables (split in quartiles). The associations between cytokine levels with the study outcomes were analyzed in two multivariable models adjusting for different sets of risk factors: (1) demographic factors (age, sex) and (2) demographic factors and baseline vascular factors (hypertension, diabetes, current smoking, history of atrial fibrillation, anticoagulants, antihypertensive medications, antiplatelet agents, statins, and LDL cholesterol).

To address the missing values in LDL cholesterol under the assumption of missing at random, we employed multiple imputation by chained equations (MICE) with predictive mean matching (PMM; five imputations, ten iterations) using the *mice* package (version 3.16.0) in R.^37^ The imputation model incorporated age, sex, hypertension, diabetes, current smoking, history of stroke, statin medication use, and BMI to predict LDL cholesterol values. All Cox regression models were executed separately within each imputed dataset. Subsequently, regression coefficients and standard errors were pooled according to Rubin’s Rules^38^ to derive combined adjusted hazard ratios (aHR), 95% confidence intervals (CI), and corresponding *p* values for each adjustment model. FDR correction was applied to control for multiple comparisons in these analyses.

Sensitivity analyses were conducted to examine the robustness of the findings. Specifically, analyses were repeated after excluding patients with hemorrhagic stroke (*n* = 12) from the initial study cohort, as well as after excluding TIA cases from the main outcomes. To explore whether cytokine–recurrence associations differed by ischemic stroke mechanism, we fitted Cox models separately within each TOAST ischemic subtype for the two cytokines showing significant associations with recurrence. For each subtype we required ⩾5 recurrent events; subtypes with fewer events were omitted due to unstable estimates. We tested for heterogeneity in associations across subtypes using likelihood ratio tests. To assess whether carotid anatomy influenced our findings, we performed a post-hoc sensitivity analysis by deriving a dichotomous significant carotid stenosis variable – defined as ⩾70% narrowing or occlusion in at least one internal carotid artery on Doppler/ultrasound – and repeating the fully adjusted Cox models for recurrent stroke/TIA and composite vascular events, restricting to participants with available stenosis data. Kaplan–Meier curves were generated to visualize time-to-events across different cytokine quartiles, and differences between groups were compared using the log-rank test.

Model discrimination was assessed by calculating Harrell’s C-index across all imputed datasets. For each outcome, C-indices were pooled according to Rubin’s rules^38^ to account for both within-imputation and between-imputation variance. Incremental discriminative value of inflammatory biomarkers was evaluated by calculating ΔC-index between nested models, with statistical significance determined using the *cindex.comp* function. Models were constructed hierarchically, beginning with basic clinical variables (age and sex), followed by addition of either CD62E, MIF, or both biomarkers simultaneously, with further adjustment for established vascular risk factors and C-reactive protein. Both cfNRI and IDI were calculated using the exact same hierarchical, nested model sequence as for the C-index, evaluated at 5 years with 95%CIs and two-sided *p* values.

## Results

### Study flowchart and baseline characteristics

**Figure 1.**
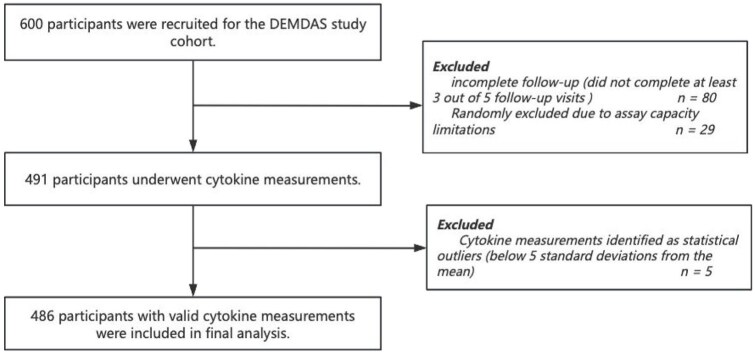
Flowchart of the participants inclusion and exclusion criteria.

DEMDAS recruited 600 participants. Due to limited reagent availability, plasma levels of 22 cytokines could only be quantified in a subset of the total study cohort. To ensure sufficient longitudinal data for robust analysis, we prioritized participants who completed at least three assessments during the 5-year follow-up (*n* = 520). From this group, we randomly selected 491 participants for cytokine measurements, minimizing potential selection bias. Five participants were excluded because more than 80% of their cytokine measurements were classified as outliers, defined as values lower than 5 SD below the mean. As a result, 486 participants were included in the final analysis (Figure 1). Their baseline characteristics are summarized in Table 1. The baseline characteristics of included participants did not differ significantly from those of participants who were not included in the analysis (Supplementary Table S1). The included participants had a median age of 68 years (IQR, 60–76 years) and 33.7% were female. Supplementary Table S2 provides an overview of the 22 cytokines measured in this study, including their full names, abbreviations, and biological functions.

**Table 1.** Baseline characteristics of participants in the DEMDAS study included in the current analysis.

Baseline characteristic	Overall (*N* = 486)
Age at recruitment (years), median (Q1, Q3)	68.0 (60.0, 76.0)
Female sex, *n* (%)	164 (33.7%)
BMI (kg/m^2^), median (Q1, Q3)	26.5(24.3, 29.1)
LDL cholesterol (mg/dL), median (Q1, Q3)	124.0 (101.0, 151.6)
C-reactive protein (mg/dL), median (Q1, Q3)	0.3 (0.1, 0.6)
Missing	135
NIHSS score at baseline, median (Q1, Q3)	2 (1, 5)
Diabetes, *n* (%)	91 (19%)
Hypertension, *n* (%)	370 (76%)
Atrial fibrillation, *n* (%)	94 (19%)
Previous stroke, *n* (%)	49 (10%)
Current smoking, *n* (%)	123 (25%)
Current drinking, *n* (%)	363 (75%)
Stroke classification	
Ischemic stroke (TOAST subtypes), *n* (%)	474 (97.5%)
Large artery atherosclerosis	133 (27%)
Cardioembolism	105 (22%)
Small artery occlusion	57 (12%)
Other etiology	25 (5.1%)
Undefined etiology	154 (32%)
Hemorrhagic stroke	12 (2.5%)
Stroke lesion volume (mm^3^), median (Q1, Q3)	2344 (512, 13,048)
Missing	18
Normalized stroke lesion volume (%), median (Q1, Q3)	0.2 (0.0, 0.8)
Missing	22

BMI: body mass index; LDL: low-density lipoprotein; NIHSS: National Institutes of Health Stroke Scale; TOAST: Trial of Org 10172 in Acute Stroke Treatment; IQR: interquartile range.

### Determinants of baseline cytokine levels

**Figure 2.**
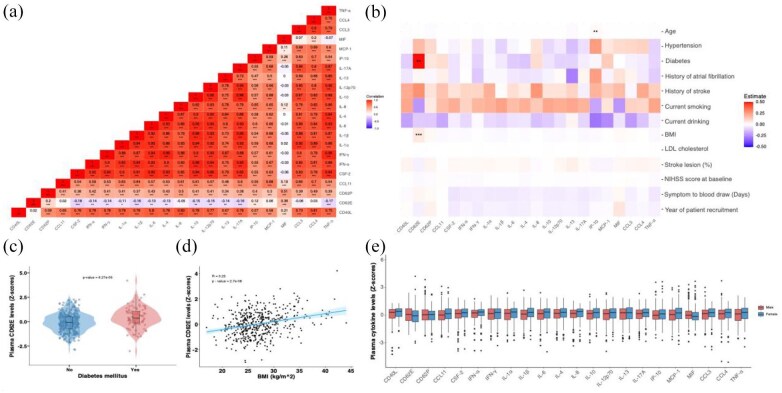
Correlations across cytokine levels and the effects of demographic, clinical, and technical factors on cytokine levels. (a) Heatmap illustrating the Spearman correlations across the plasma levels of 22 cytokines in the DEMDAS cohort (*N* = 486). The color scale ranges from blue (negative correlation) to red (positive correlation). Significance is indicated by *p < 0.05, **p < 0.01, and ***p < 0.001, representing FDR – corrected *p* values for multiple comparisons. (b) Heatmap illustrating the effect estimates of demographic, clinical, and technical factors on ln-transformed standardized cytokine levels, as derived from univariable linear regression analyses. Red and blue colors represent positive and negative estimates, respectively. Significance is indicated by *p < 0.05, **p < 0.01, and ***p < 0.001 (all FDR-adjusted). (c) Violin plot illustrating the difference in plasma CD62E levels between patients with and without diabetes mellitus. The box represents the IQR (25th–75th percentiles), with the line inside the box representing the median. (d) Scatter plot illustrating the positive correlation between plasma CD62E levels and BMI. (e) Box plot illustrating comparisons in the sex-specific distribution of cytokine levels across the DEMDAS cohort. Each box represents IQR, covering the 25th–75th percentiles of the data, with the line inside the box indicating the median value. The whiskers extend to the most extreme data points within 1.5 times the IQR. BMI: body mass index; FDR: false discovery rate; LDL: low density lipoprotein; NIHSS: National Institutes of Health Stroke Scale; IQR: interquartile range; FDR: false discovery rate.

The distributions of ln-transformed, standardized values for the 22 quantified cytokines are illustrated in Supplementary Figure S1. Most cytokines showed similar patterns of variation, except for CD62E and MIF, which did not closely align with the patterns of other cytokines (Figure 2(a) and Supplementary Table S3). The median time between stroke onset and blood draw was 3.03 days (IQR 1.98–4.41 days) and more than 85% of the samples were fasting samples collected in the morning. Univariable linear regression analyses showed that most demographic, clinical, and technical factors had no significant associations with cytokine levels (Figure 2(b) and Supplementary Table S4). Most importantly, technical factors, including time from symptom onset to blood draw, as well as the year of patient recruitment (a proxy of the time that the sample remained frozen before cytokine quantification) were not associated with plasma levels of any of the cytokines (Figure 2(b)). Among vascular risk factors, we found a positive correlation between CD62E levels and BMI (Spearman’s ρ = 0.25, *p* < 0.001), as well as higher CD62E levels in patients with diabetes (Figure 2(b)–(d)). There were no significant differences in the levels of any of the 22 cytokines between male and female study participants (Figure 2(e) and Supplementary Table S5).

### Associations between cytokine levels and recurrent events

**Figure 3.**
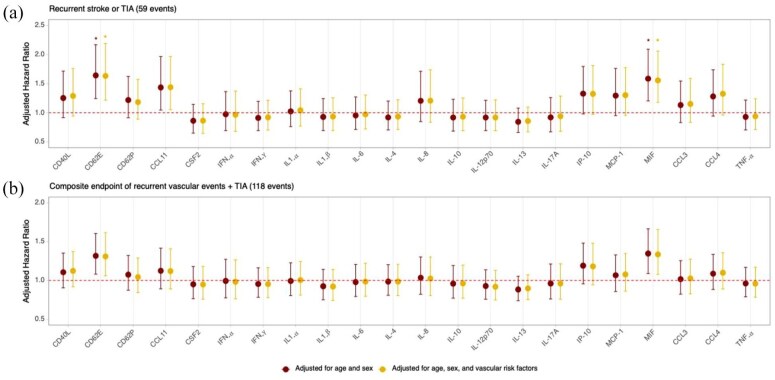
Associations of cytokine levels with recurrent stroke or TIA and recurrent vascular events (stroke, TIA, acute coronary syndrome, new onset of heart failure, or death) over the 5-year follow-up period. Forest plots of adjusted HR for the associations of circulating baseline cytokine levels (per SD increment in ln-transformed values) with (a) recurrent stroke or TIA (*n* = 59) and (b) the composite endpoint of recurrent vascular events (*n* = 118) over the 5-year follow-up period. The HR in the first model (red) are adjusted for age and sex, while those in the second model (yellow) are further adjusted for vascular risk factors, including hypertension, diabetes, current smoking, history of atrial fibrillation, anticoagulants, antihypertensive medications, antiplatelet agents, statins, and low-density lipoprotein levels. Vertical error bars indicate the 95%CI for each adjusted HR. Significance is indicated by *FDR adjusted *p* < 0.05, representing FDR-corrected *p* values for multiple comparisons. TIA: transient ischemic attack; HR: hazard ratio; CI: confidence interval; FDR: false discovery rate.

During the 5-year follow-up period, 59 (12.1%) participants experienced a recurrent stroke or TIA, and 118 (24.3%) participants experienced a composite of recurrent vascular events, including stroke, TIA, acute coronary syndrome, acute or decompensated heart failure, or death. Figure 3 illustrates the aHR for the associations between the levels of the 22 cytokines (per SD increment in ln-transformed values) and recurrent stroke or TIA (Figure 3(a) and Supplementary Table S6) as well as recurrent vascular events (Figure 3(b) and Supplementary Table S7), derived from Cox regression analyses adjusted for age, sex, and vascular risk factors. Plasma levels of CD62E and MIF were associated with a higher risk of recurrent stroke or TIA over the 5-year follow-up period (FDR-adjusted *p* = 0.01) (Figure 3(a) and Supplementary Table S6). These associations remained significant when adjusting for age and sex (aHR for CD62E: 1.642 (95%CI 1.244–2.168); aHR for MIF: 1.586 (95%CI 1.203–2.091)) and also when additionally adjusting for vascular risk factors (hypertension, diabetes, current smoking, history of atrial fibrillation, anticoagulants, antihypertensive medications, antiplatelet agents, statins, and low-density lipoprotein levels; aHR for CD62E: 1.632 (95%CI 1.216–2.190); aHR for MIF: 1.557 (95%CI 1.177–2.059)). Although the associations between CD62E and MIF and recurrent vascular events also met the nominal threshold of *p* < 0.05, they were not significant after FDR correction (Figure 3(b) and Supplementary Table S7). Sensitivity analyses, excluding patients with hemorrhagic stroke (*n* = 12) at baseline (Supplementary Figure S2 and Supplementary Tables S8 and S9) and excluding TIA from the outcomes of interest (Supplementary Figure S3 and Supplementary Tables S10 and S11) yielded similar with consistent effects for both CD62E and MIF. Moreover, in a post-hoc sensitivity analysis adding high-grade carotid stenosis (⩾70%) to the fully adjusted models, the associations of CD62E and MIF with outcomes remained robust (Supplementary Table S12). Stratifying by TOAST ischemic subtype, we observed significant heterogeneity for MIF across subtypes (*p* = 0.046) and borderline significant heterogeneity for CD62E (*p* = 0.059). Both cytokines showed significant associations with recurrence only in the undetermined subgroup (*N* = 154; events = 21): MIF (aHR: 2.29, 95%CI 1.51–3.45; FDR-adjusted *p* = 0.001) and CD62E (aHR: 2.24, 95%CI 1.38–3.63; FDR-adjusted *p* = 0.005; Supplementary Table S13).

**Figure 4.**
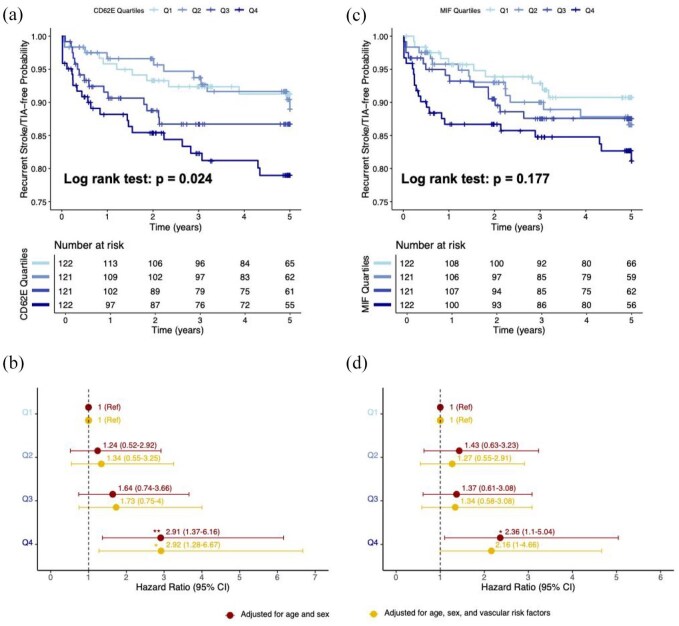
Dose–response associations of baseline CD62E and MIF levels with 5-year risk of recurrent stroke or TIA. (a) Kaplan–Meier 5-year recurrent stroke- or TIA-free survival curves for DEMDAS participants across quartiles (Q1–Q4) of baseline circulating CD62E levels. (b) Forest plot of adjusted HR for recurrent stroke or TIA across quartiles of baseline circulating CD62E levels. (c) Kaplan–Meier 5-year recurrent stroke- or TIA-free survival curves for DEMDAS participants across quartiles (Q1–Q4) of baseline circulating MIF levels. (d) Forest plot of adjusted HR for recurrent stroke or TIA across quartiles of baseline circulating MIF levels. In panels (b) and (d), the HR in the first model (red) are adjusted for age and sex, while the HR in the second model (yellow) are further adjusted for vascular risk factors, including hypertension, diabetes, current smoking, history of atrial fibrillation, anticoagulants, antihypertensive medications, antiplatelet agents, statins, and low-density lipoprotein levels. Horizontal lines represent the 95%CI for each HR. Statistical significance is indicated by *p < 0.05, **p < 0.01. TIA: transient ischemic attack; HR: hazard ratio; CI: confidence interval.

When analyzing by quartiles of the baseline CD62E and MIF distributions, dose dependent increases in the risk of recurrent stroke or TIA were observed for both proteins (Figure 4). Compared with the first quartile (Q1), patients in the highest quartiles of CD62E and MIF distributions (Q4) were at statistically significantly higher risk of recurrent stroke or TIA over the 5-year follow-up period (aHR for CD62E Q4 vs Q1: 2.906 (95%CI 1.370–6.162); aHR for MIF Q4 vs Q1: 2.357 (95%CI 1.102–5.043)). These effects remained robust after adjusting for age, sex, and vascular risk factors, as well as when excluding TIA from the definition of the outcome (Supplementary Figure S4). For CD62E, similar albeit weaker associations were noted for the composite endpoint of recurrent vascular events (Supplementary Figure S5).

### Predictive performance of CD62E and MIF for recurrent events

As a final step, we assessed whether adding baseline CD62E and MIF levels to models including baseline demographic and vascular risk factors, as well as CRP levels, could improve the prediction of recurrent stroke or TIA (Table 2). The addition of baseline CD62E and MIF levels not only improved the C-index of Cox regression models for 5-year risk of recurrent stroke or TIA on top of age and sex (ΔC-index = 0.050, *p* = 0.046), age, sex, and vascular risk factors (ΔC-index = 0.038, *p* = 0.041), as well as age, sex, vascular risk factors, and baseline CRP levels (ΔC-index = 0.044, *p* = 0.012), but also yielded positive reclassification and discrimination gains. In continuous NRI analyses, cfNRI was 0.195 (95%CI 0.015–0.323; *p* = 0.032), 0.169 (95%CI −0.004 to 0.322; *p* = 0.056), and 0.213 (95%CI 0.013–0.400; *p* = 0.038) for those same three comparisons, while IDI was 0.044 (95%CI 0.011–0.123; *p* < 0.001), 0.043 (95%CI 0.011–0.124; *p* = 0.004), and 0.074 (95%CI 0.018–0.172; *p* = 0.002), respectively (Table 2). Similar improvements were observed when analyzing 5-year risk of recurrent stroke, excluding TIA cases (Supplementary Table S14).

**Table 2.** Performance comparisons of predictive models for 5-year risk of recurrent stroke or TIA, incorporating different combinations of age, sex, VRF (hypertension, diabetes, current smoking, history of atrial fibrillation, anticoagulants, antihypertensive medications, antiplatelet agents, statins, and LDL cholesterol), and baseline circulating levels of CRP, CD62E, and MIF – showing C-index (95%CI), change in C-index (ΔC-index), cfNRI (95%CI), and IDI (95%CI).

*Model*	*C-index (95%CI)*	*ΔC-index*	*ΔC-index_p*	*cfNRI (95%CI)*	*cfNRI_p*	*IDI (95%CI)*	*IDI_p*
Age + sex	0.616 (0.544–0.687)	Ref.	—				
Age + sex + CD62E	0.661 (0.593–0.729)	0.045	0.067	0.109 (−0.000 to 0.265)	0.052	0.033 (0.005–0.095)	0.006
Age + sex + MIF	0.646 (0.579–0.714)	0.031	0.110	0.051 (−0.077 to 0.222)	0.344	0.027 (0.004–0.099)	0.002
Age + sex + CD62E + MIF	0.666 (0.599–0.733)	0.050	0.046	0.195 (0.015–0.323)	0.032	0.044 (0.011–0.123)	0.000
Age + sex + VRF	0.657 (0.591–0.723)	Ref.	—				
Age + sex + VRF + CD62E	0.689 (0.622–0.757)	0.032	0.074	0.099 (−0.031 to 0.260)	0.120	0.033 (0.005–0.104)	0.006
Age + sex + VRF + MIF	0.688 (0.620–0.756)	0.030	0.047	0.042 (−0.085 to 0.197)	0.396	0.026 (0.003–0.089)	0.016
Age + sex + VRF + CD62E + MIF	0.696 (0.629–0.763)	0.038	0.042	0.169 (−0.004 to 0.322)	0.056	0.043 (0.011–0.124)	0.004
Age + sex + VRF + CRP^a^	0.712 (0.645–0.788)	Ref.	—				
Age + sex + VRF + CRP^a^ + CD62E	0.743 (0.674–0.811)	0.031	0.056	0.163 (−0.047 to 0.348)	0.132	0.055 (0.004–0.150)	0.020
Age + sex + VRF + CRP^a^ + MIF	0.754 (0.687–0.820)	0.042	0.005	0.144 (−0.070 to 0.317)	0.186	0.048 (0.005–0.124)	0.024
Age + sex + VRF + CRP^a^ + CD62E + MIF	0.756 (0.689–0.823)	0.044	0.012	0.213 (0.013–0.400)	0.038	0.074 (0.018–0.172)	0.002

TIA: transient ischemic attack; VRF: vascular risk factors; LDL: low-density lipoprotein; cfNRI: category free net reclassification index; CRP: C-reactive protein; CI: confidence interval; Ref.: reference; IDI: integrated discrimination improvement.

^a^Analysis restricted to 343 individuals with available CRP levels (41 recurrent stroke or TIA events).

## Discussion

In this multicenter prospective cohort study of 486 acute stroke patients, we aimed to comprehensively evaluate the predictive value of a panel of 22 cytokines for risk of vascular recurrence. We found that higher baseline levels of CD62E and MIF were associated with an increased risk of recurrent stroke or TIA over a 5-year follow-up period. These associations were robust to adjustments for baseline demographic and vascular risk factors, including in a post-hoc sensitivity analysis adding significant carotid stenosis (⩾70%), and followed dose-response patterns. Adding baseline CD62E and MIF levels on top of age, sex, vascular risk factors, and baseline CRP levels led to significant improvements in risk discrimination for recurrent stroke or TIA (ΔC-index: 0.030–0.050) and achieved positive reclassification and discrimination gains (cfNRI up to 0.213; IDI up to 0.074).

Our findings may have important implications for risk stratification in stroke patients, particularly in selecting candidates for anti-inflammatory treatments. In line with the CANTOS trial,^17^ the ongoing ZEUS trial,^25^ which tests an anti-IL-6 antibody in a broad population with atherosclerosis (including atherosclerotic stroke), uses a CRP threshold of >2 mg/dL to select patients. Similarly, the CASPER trial, which tests colchicine in ischemic stroke patients, restricts enrollment to those with CRP levels >2 mg/dL.^39^ While a meta-analysis supports an association between CRP levels and stroke recurrence,^24^ CRP is not specific for arterial inflammation, and transient increases in the acute phase after stroke could limit its utility for risk stratification at that critical time window.^40^ Here, we provide evidence that two other inflammatory mediators, CD62E and MIF, may offer improved prediction of recurrent stroke beyond traditional risk factors and CRP levels. In our study, the point estimates for the associations between CD62E and MIF and recurrent stroke risk (CD62E: HR for Q4 vs Q1: 2.906; MIF: HR for Q4 vs Q1: 2.357) appeared larger than those reported for CRP in largest meta-analysis to date by McCabe et al. (HR for Q4 vs Q1: 1.40 when adjusting for age and sex), though such cross-study comparisons must be interpreted cautiously.^24^ Furthermore, these CRP associations became barely significant following adjustments for vascular risk factors (HR: 1.05, *p* = 0.058), whereas CD62E and MIF maintained significant associations with recurrent stroke risk in our study. In our cohort, CRP showed no significant association (HR/ln-unit: 1.14, 95%CI: 0.90–1.44, *p* = 0.28), limited by substantial missing data (29%). While CD62E and MIF levels are known to be elevated in certain infections, such as infective endocarditis and brucellosis,^41–43^ their associations with recurrent stroke in our study remained significant after adjusting for CRP, suggesting their predictive value may extend beyond infection-related inflammation. Notably, IL-6 did not show significant associations with recurrent stroke/TIA in our analysis adjusted for age, sex and vascular risk factors (aHR/SD increment: 0.97, 95%CI: 0.72–1.31; *p* = 0.75), contrasting with the McCabe meta-analysis which found IL-6 to be a significant predictor of stroke recurrence (HR Q4 vs Q1: 1.33, 95%CI: 1.08–1.65). This may reflect differences in study population and statistical power. While CD62E and MIF might be more specific for vascular inflammation compared to traditional acute-phase reactants, they can still be elevated as a result of infections or systemic inflammatory conditions, particularly in the acute phase of stroke. Whether these biomarkers could complement established markers in selecting patients for anti-inflammatory treatments requires further investigation in external cohorts and in *post hoc* analysis of randomized trial data. It is noteworthy, however, that despite the significant association of the highest quartile (Q4) of MIF with recurrent stroke/TIA in adjusted Cox models, the Kaplan–Meier analysis did not demonstrate statistically significant differences among MIF quartiles (log-rank *p* = 0.177). This discrepancy likely reflects limited statistical power, emphasizing the need for validation in larger cohorts to clarify the clinical utility of MIF measurement. Subtype-stratified analyses revealed significant heterogeneity in MIF associations across stroke mechanisms (*p* = 0.046), with significant associations observed only in the cryptogenic/undetermined subgroup. However, these exploratory findings are based on a small number of patients and events within each subtype, making them difficult to interpret and requiring validation in larger, mechanism-focused cohorts specifically designed to investigate cytokine associations within stroke subtypes.

Both CD62E and MIF have been extensively linked to the pathophysiology of vascular disease. CD62E, an adhesion molecule also known as E-selectin, mediates leukocyte adhesion to the endothelium, a critical step in the recruitment of immune cells to the subendothelial space and the initiation of arterial inflammation.^44^ As inflammation progresses, CD62E undergoes increased shedding, making it detectable in plasma and reflecting its upregulation. This shedding process, mediated by metalloproteinases such as ADAM17, has been shown to regulate adhesion molecule function and contribute to inflammatory signaling pathways.^45–48^ Mouse studies have shown that targeting E-selectin reduces atherosclerotic lesions and improves heart function.^49,50^ In acute coronary syndrome, elevated CD62E levels reflect endothelial activation, promoting leukocyte adhesion – a hallmark of inflammation and vascular dysfunction.^50,51^ While studies directly linking CD62E to stroke recurrence are limited, it has been identified as a predictor of poor 3-month outcomes in stroke patients, including functional impairment.^52^ Experimental models further demonstrate that blocking E-selectin improves cerebral blood flow and reduces infarct size, highlighting its therapeutic potential.^53^ Additionally, Mendelian Randomization analyses have associated genetically proxied CD62E with adverse post-stroke outcomes, reinforcing its role in cerebrovascular disease.^54^ Similarly, MIF – a pro-inflammatory cytokine and atypical chemokine,^55^ drives the atherogenic recruitment of monocytes, neutrophils, T cells, and platelets to atherosclerotic plaques and has been implicated in plaque instability and rupture.^56–59^ Targeting MIF has been shown to reduce atherosclerosis in experimental mouse models.^57,60–63^ A study of 469 stroke patients with a shorter follow-up (12 months) also found significant associations between baseline MIF levels and risk of recurrent stroke.^64^ In another study of stroke survivors, elevated MIF levels in the acute phase after stroke were linked to poorer stroke outcomes.^65^

Our study has several limitations. First, cytokine levels were measured only once in the acute phase (median 3 days post-stroke). This limits our ability to assess intra-patient variability or temporal dynamics, which have been raised as concerns for traditional markers such as CRP and IL-6. While CD62E and MIF may offer greater specificity for vascular inflammation, they remain susceptible to elevation from systemic infections or inflammatory conditions. Moreover, we did not systematically exclude patients with such conditions. Dedicated studies with serial cytokine measurements and careful control of inflammatory confounders are needed to clarify the added value of these biomarkers. Second, the relatively modest sample size of the DEMDAS cohort (*N* = 600), as predetermined by the original study protocol,^34^ limited the statistical power to detect more subtle associations, particularly in stratified or subtype-specific analyses. In addition, a 15% attrition rate over the 5-year follow-up may have led to underestimation of recurrence rates and introduced attrition bias. This was an exploratory analysis outside the original DEMDAS protocol, which focused on risk factors of post-stroke dementia. The demanding follow-up – requiring serial MRI and neuropsychological testing – limited participation and retention, a common challenge in longitudinal neuroimaging studies.^5,66^ Importantly, baseline demographic and clinical characteristics of patients who completed the study versus those lost to follow-up were broadly similar, mitigating some concerns about attrition bias. Third, the intensity of the study protocol also influenced the initial recruitment process, resulting in preferential enrollment of patients with milder strokes who were more likely to adhere with repeated imaging and cognitive assessments.^29,33^ This overrepresentation of less severely affected individuals likely contributed to relatively lower observed recurrence rates (~12%) which further reduced statistical power and may have obscured additional cytokine associations despite FDR correction. Consequently, all cytokine associations identified here should be considered exploratory and require validation in larger, more representative cohorts. Although this might limit the generalizability of our findings to a broader stroke population, these patients may represent a group that could benefit from more aggressive secondary prevention including anti-inflammatory therapies. Fourth, CRP levels were missing in a substantial proportion of our study population (29%), further limiting statistical power when comparing CD62E and MIF levels with CRP. Fifth, because of limited data on the exact causes of death, our definition of recurrent vascular events included all-cause mortality rather than only vascular death, deviating from the standard definition of major adverse cardiovascular events.^67^ However, since cardiovascular disease is the most common cause of death among stroke survivors^68^ and since there were only 46 deaths over the 5-year follow-up period, we do not expect this to have introduced substantial misclassification bias. Sixth, although all outcome events were verified through medical record review and imaging by physicians, initial reporting relied on patient or informant accounts during structured follow-up interviews. Therefore, the possibility of misclassification due to recall or reporting bias cannot be entirely excluded.

In conclusion, our study demonstrates that elevated plasma levels of CD62E and MIF shortly after acute stroke are associated with a higher 5-year risk of recurrent stroke or TIA, offering improved predictive value on top of traditional risk factors and CRP levels. However, these findings are preliminary and need to be confirmed through larger studies to establish their clinical relevance. If validated in future studies, assessing these cytokines may complement existing approaches to risk stratification for stroke patients and inform patient selection for randomized trials of anti-inflammatory agents.

## Supplementary Material

sj-docx-1-eso-23969873251360145

sj-xlsx-2-eso-23969873251360145

## Trial registration

The study is registered at http://www.clinicaltrials.gov (NCT01334749).
