# Correction to: Circulating cytokine levels and 5-year vascular recurrence after stroke: A multicenter prospective cohort study

**DOI:** 10.1093/esj/aakag101

**Published:** 2026-08-18

This is a correction to: Lanyue Zhang, Mohamad Ali Antabi, Jana Mattar, Omar El Bounkari, Rong Fang, Karin Waegemann, Felix J Bode, Sebastian Stösser, Peter Hermann, Thomas G Liman, Christian H Nolte, Benno Ikenberg, Kathleen Bernkopf, Wenzel Glanz, Daniel Janowitz, Annika Spottke, Michael Wolfgang Görtler, Silke Wunderlich, Inga Zerr, Gabor C Petzold, Matthias Endres, Jürgen Bernhagen, Martin Dichgans, Marios K Georgakis, DEMDAS Investigators, Circulating cytokine levels and 5-year vascular recurrence after stroke: A multicenter prospective cohort study, *European Stroke Journal*, Volume 11, Issue 1, January 2026, 23969873251360145, https://doi.org/10.1093/esj/23969873251360145

In the OUP publication of the article, the author list and details have been corrected in the non-pdf version to align with that of the PDF. The author list now reads: “Lanyue Zhang, Mohamad Ali Antabi, Jana Mattar, Omar El Bounkari, Rong Fang, Karin Waegemann, Felix J Bode, Sebastian Stösser, Peter Hermann, Thomas G Liman, Christian H Nolte, Benno Ikenberg, Kathleen Bernkopf, Wenzel Glanz, Daniel Janowitz, Annika Spottke, Michael Wolfgang Görtler, Silke Wunderlich, Inga Zerr, Gabor C Petzold, Matthias Endres, Jürgen Bernhagen, Martin Dichgans, Marios K Georgakis, DEMDAS Investigators” instead of: “Lanyue Zhang, Mohamad Ali Antabi, Jana Mattar, Omar El Bounkari, Rong Fang, Karin Waegemann, Felix J Bode, Sebastian Stösser, Peter Hermann, Thomas G Liman, Christian H Nolte, Benno Ikenberg, Kathleen Bernkopf, Gabor C Petzold, Jürgen Bernhagen, Martin Dichgans, Marios K Georgakis, DEMDAS Investigators”.

