# Supplementary material for: Circulating cytokine levels and 5-year vascular recurrence after stroke: A multicenter prospective cohort study
Source: Eur Stroke J. 2026 Jan 1;11(1):23969873251360145. doi: 10.1093/esj/23969873251360145 (PMC12866279; doi:10.1093/esj/23969873251360145)
Supplement: supplementary_files_23969873251360145 [file supplementary_files_23969873251360145.zip › sj-docx-1-eso-23969873251360145.docx]

**Supplemental Figures and Figure Legends**

**Circulating cytokine levels and 5-year vascular recurrence after stroke:**

**a multicenter prospective cohort study**

Lanyue Zhang^1^*, Mohamad Ali Antabi^1^*, Jana Mattar^1^, Omar El Bounkari^1^, Rong Fang^1^, Karin Waegemann^1, 2^, Felix J. Bode^3, 4^, Sebastian Stösser^3, 4^, Peter Hermann^5, 6^, Thomas G. Liman^7-9^, Christian H. Nolte^8, 10, 11^, Benno Ikenberg^12^, Kathleen Bernkopf^12^, Wenzel Glanz^13, 14^, Daniel Janowitz^1^, Annika Spottke^3,4^, Michael Görtler^13,14^, Silke Wunderlich^12^, Inga Zerr^5, 6^; Gabor C. Petzold^3, 4^, Matthias Endres^7, 8, 11, 16,17^, Jürgen Bernhagen^1^,^2,19^, Martin Dichgans^1, 2,18,19^, Marios K. Georgakis^1,19, 20^ on behalf of the DEMDAS investigators^#^

^1^ Institute for Stroke and Dementia Research (ISD), LMU University Hospital, LMU Munich, Munich, Germany

^2^ German Center for Neurodegenerative Diseases (DZNE), Munich, Germany

^3^ German Center for Neurodegenerative Diseases (DZNE), Bonn, Germany

^4^ Department of Vascular Neurology, University Hospital Bonn, Bonn, Germany

^5^ Department of Neurology, University Medical Center Göttingen, Göttingen, Germany

^6^ German Center for Neurodegenerative Diseases (DZNE), Göttingen, Germany

^7^ Center for Stroke Research Berlin (CSB), Charité - Universitätsmedizin Berlin

^8^ German Center for Neurodegenerative Diseases (DZNE), Berlin, Germany

^9^ Department of Neurology, Carl Von Ossietzky University, Oldenburg, Germany

^10^ Berlin Institute of Health (BIH), Germany

^11^ Department of Neurology with Experimental Neurology, Charité - Univeristätsmedizin Berlin

^12^ Department of Neurology, Technical University of Munich, School of Medicine and Health, Klinikum rechts der Isar, München, Germany

^13^ Department of Neurology, University Hospital, Otto-von-Guericke University Magdeburg, Magdeburg, Germany

^14^ German Center for Neurodegenerative Diseases (DZNE), Magdeburg, Germany

^15^ Department of Old Age Psychiatry and Cognitive Disorders, University Hospital Bonn, Bonn, Germany

^16^ German Centre for Cardiovascular Research (DZHK), partner site Berlin, Berlin, Germany

^17^ German Center for Mental Health (DZPG), partner site Berlin, Berlin, Germany

^18^ German Centre for Cardiovascular Research (DZHK), Munich, Germany

^19^ Munich Cluster for Systems Neurology (SyNergy), Munich, Germany

^20^ Program in Medical and Population Genetics and Cardiovascular Disease Initiative, Broad Institute of MIT and Harvard, Cambridge, MA, USA

* These authors contributed equally to this study

^#^ Names and affiliations of DEMDAS investigators are listed in the supplement (**Table S12**)

**Corresponding author**

Marios K. Georgakis, MD, PhD

Institute for Stroke and Dementia Research

Ludwig-Maximilians-University Hospital

Feodor-Lynen-Str. 17, 81377 Munich, Germany

Tel: +49-89-4400-46127, Fax: +49-89-4400-46040

E-mail: [marios.georgakis@med.uni-muenchen.de](mailto:marios.georgakis@med.uni-muenchen.de)

**Supplementary Figure S1. Boxplot and density of the baseline plasma levels of the 22 quantified cytokines.** The X-axis represents the Ln-transformed and batch-standardized cytokine levels (Z-scores), while the Y-axis displays the corresponding cytokines. The boxplots illustrate the interquartile range (IQR; 25th to 75th percentiles), with the line within each box indicating the median.

**Supplementary Figure S2. Associations between cytokine levels and recurrent stroke/TIA and recurrent vascular events (stroke, acute coronary syndrome, new-onset heart failure, or death) over the 5-year follow-up across different adjustment models in ischemic stroke patients**. Forest plots of adjusted hazard ratio (aHR) for the associations of circulating baseline cytokine levels (per standard deviation increment in ln-transformed values) with **(A)** recurrent stroke/TIA (n=57) and **(B)** the composite endpoint of recurrent vascular events with TIA (n=115) over the 5-year follow-up period. The aHR in the first model (red) are adjusted for age and sex, while those in the second model (yellow) are further adjusted for vascular risk factors, including hypertension, diabetes, current smoking, history of atrial fibrillation, anticoagulants, antihypertensive medications, antiplatelet agents, statins, and low-density lipoprotein levels. The vertical error lines indicate the 95% CI for each HR. Significance is indicated by *p-value <.05, **p-value <.01, representing FDR-corrected p-values for multiple comparisons. Abbreviations: TIA, transient ischemic attack; aHR, adjusted hazard ratio; CI, confidence interval; FDR, false discovery rate.

**Supplementary Figure S3. Associations of cytokine levels with recurrent stroke without TIA and recurrent vascular events without TIA (stroke, acute coronary syndrome, new onset of heart failure, or death) over the 5-year follow-up period**. Forest plots of adjusted hazard ratio (aHR) for the associations of circulating baseline cytokine levels (per standard deviation increment in ln-transformed values) with **(A)** recurrent stroke (n=43) and **(B)** the composite endpoint of recurrent vascular events without TIA (n=104) over the 5-year follow-up period. The HR in the first model (red) are adjusted for age and sex, while those in the second model (yellow) are further adjusted for vascular risk factors, including hypertension, diabetes, current smoking, history of atrial fibrillation, anticoagulants, antihypertensive medications, antiplatelet agents, statins, and low-density lipoprotein levels. The vertical error lines indicate the 95% CI for each HR. Significance is indicated by *p-value <.05, representing FDR-corrected p-values for multiple comparisons. Abbreviations: TIA, transient ischemic attack; aHR, adjusted hazard ratio; CI, confidence interval; FDR, false discovery rate.

**
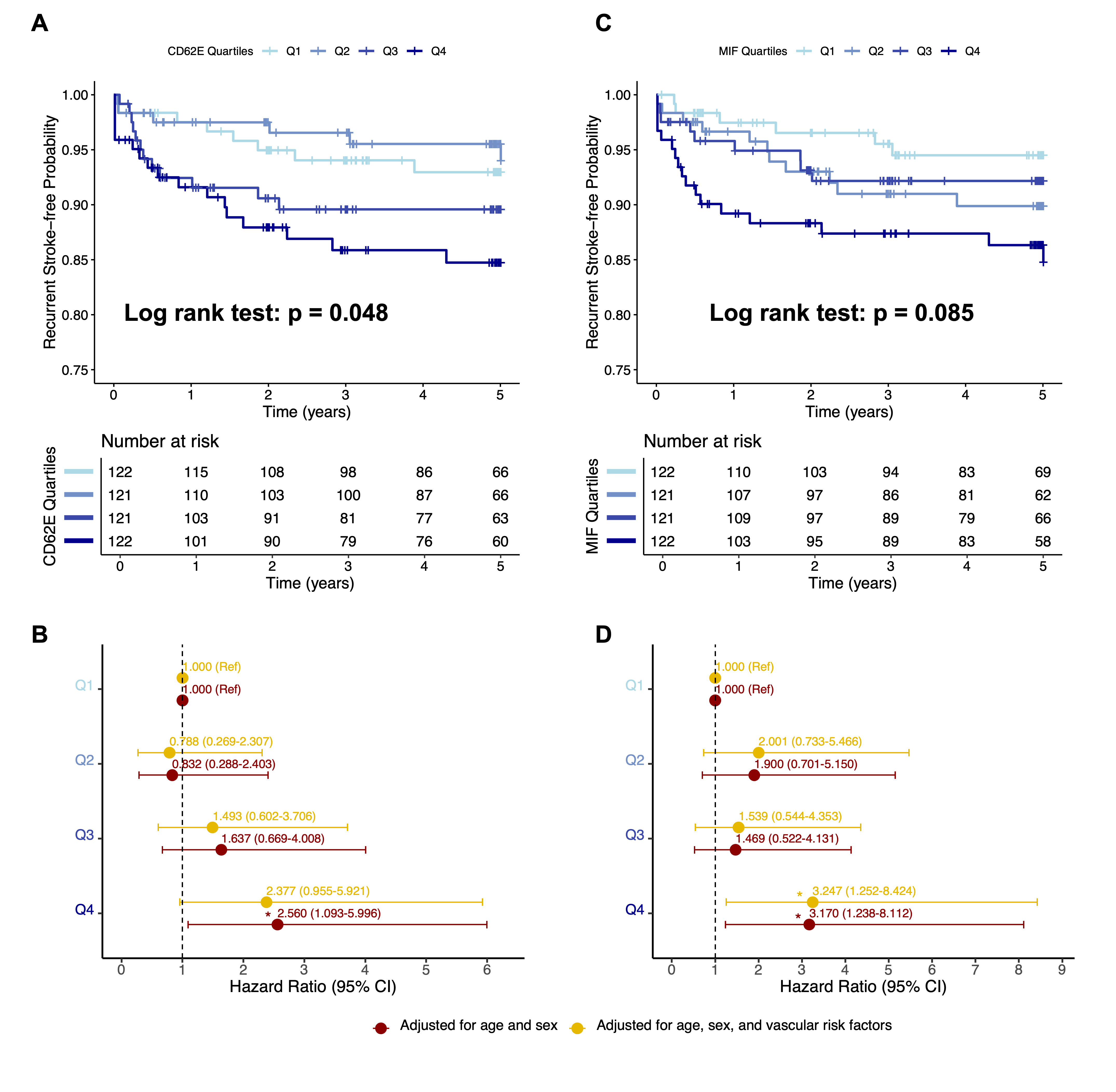
**

**Supplementary Figure S4. Dose-response associations of baseline CD62E and MIF levels with 5-year risk of recurrent stroke without TIA**. **(A)** Kaplan-Meier 5-year recurrent stroke-free survival curves for DEMDAS participants across quartiles (Q1 to Q4) of baseline circulating CD62E levels. **(B)** Forest plot of adjusted HRs for recurrent stroke across quartiles of baseline circulating CD62E levels. **(C)** Kaplan-Meier 5-year recurrent stroke-free survival curves for DEMDAS participants across quartiles (Q1 to Q4) of baseline circulating MIF levels. **(D)** Forest plot of adjusted HR for recurrent stroke across quartiles of baseline circulating MIF levels. In panels B and D, the HR in the first model (red) are adjusted for age and sex, while the HR in the second model (yellow) are further adjusted for vascular risk factors, including hypertension, diabetes, current smoking, history of atrial fibrillation, anticoagulants, antihypertensive medications, antiplatelet agents, statins, and low-density lipoprotein levels. Horizontal lines represent the 95% CI for each HR. Statistical significance is indicated by *p-value < .05, **p-value < .01. Abbreviations: TIA, transient ischemic attack; HR, hazard ratio; CI, confidence interval.


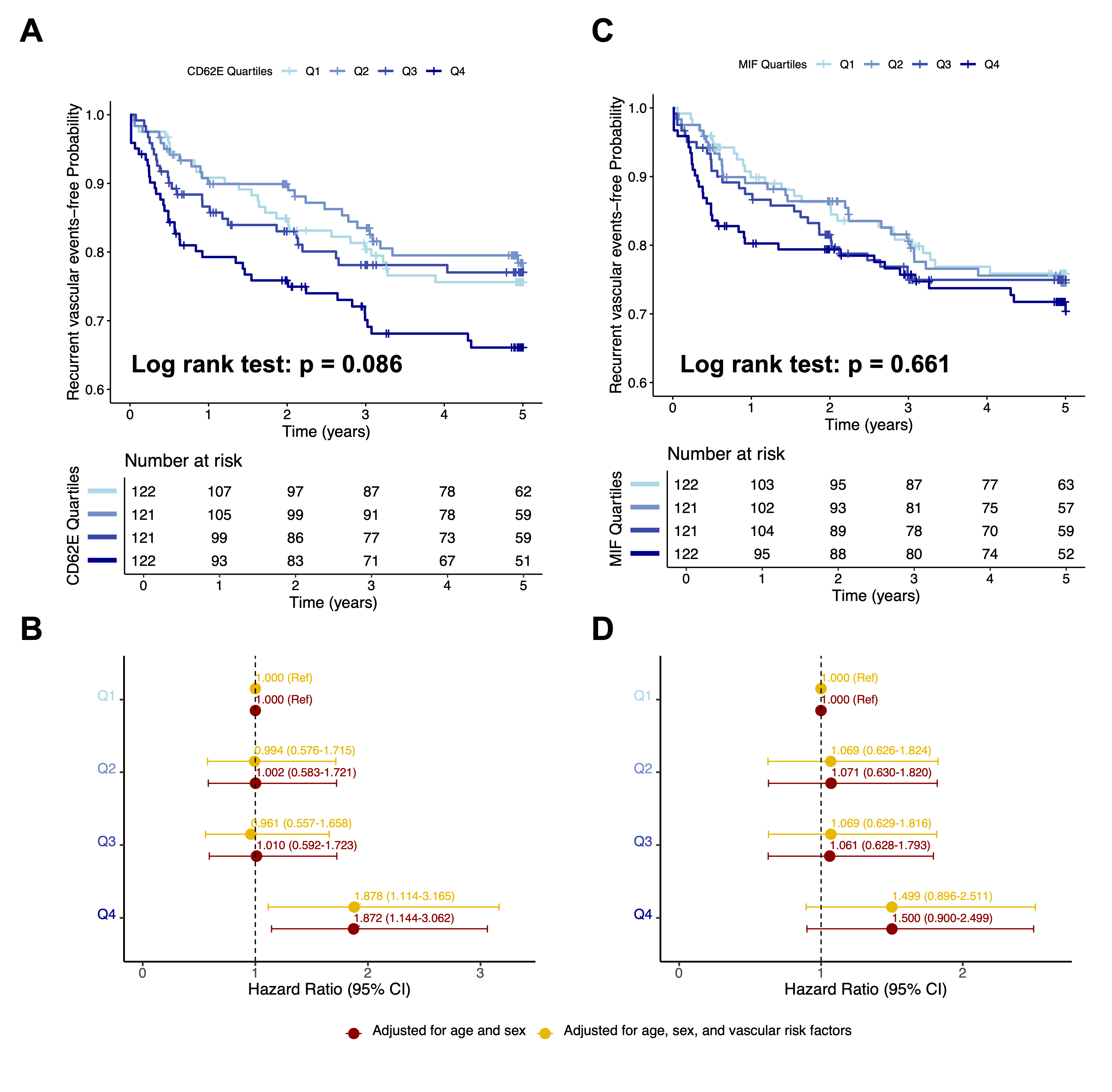


**Supplementary Figure S5**. **Dose-response associations of baseline CD62E and MIF levels with 5-year risk of recurrent vascular events (stroke, TIA, acute coronary syndrome, new onset of heart failure, or death). (A)** Kaplan-Meier 5-year recurrent vascular events-free survival curves for DEMDAS participants across quartiles (Q1 to Q4) of baseline circulating CD62E levels. **(B)** Forest plot of adjusted HR for recurrent vascular events across quartiles of baseline circulating CD62E levels. **(C)** Kaplan-Meier 5-year recurrent vascular events-free survival curves for DEMDAS participants across quartiles (Q1 to Q4) of baseline circulating MIF levels. **(D)** Forest plot of adjusted HR for recurrent vascular events across quartiles of baseline circulating MIF levels. In panels **(B)** and **(D)**, the HR in the first model (red) are adjusted for age and sex, while the HR in the second model (yellow) are further adjusted for vascular risk factors, including hypertension, diabetes, current smoking, history of atrial fibrillation, anticoagulants, antihypertensive medications, antiplatelet agents, statins, and low-density lipoprotein levels. Horizontal lines represent the 95% CI for each HR. Significance is indicated by *p-value< .05. Abbreviations: TIA, transient ischemic attack; HR, hazard ratio; CI, confidence interval.
